# Consecutive Nights of Moderate Sleep Loss Does Not Affect Mood in Healthy Young Males

**DOI:** 10.3390/clockssleep3030031

**Published:** 2021-08-20

**Authors:** Christiana Harous, Gregory D. Roach, Thomas G. Kontou, Ashley J. Montero, Nicole Stuart, Charli Sargent

**Affiliations:** 1 Appleton Institute for Behavioural Science, School of Medical, Health and Applied Sciences, Central Queensland University, 44 Greenhill Road, Wayville, Adelaide 5034, Australia; christiana.harous@flinders.edu.au (C.H.); greg.roach@cqu.edu.au (G.D.R.); t.kontou@cqu.edu.au (T.G.K.); ashley.montero@flinders.edu.au (A.J.M.); nicole.stuart@flinders.edu.au (N.S.); 2 College of Education, Psychology and Social Work, Flinders University, Sturt Road, Bedford Park, Adelaide 5042, Australia; 3 Adelaide Institute for Sleep Health, College of Medicine and Public Health, Flinders University, 5 Laffer Drive, Bedford Park, Adelaide 5049, Australia

**Keywords:** mood disturbance, emotion, wellbeing, profile of mood states, sleep restriction, fatigue

## Abstract

Sleep loss causes mood disturbance in non-clinical populations under severe conditions, i.e., two days/nights of sleep deprivation or a week of sleep restriction with 4–5 h in bed each night. However, the effects of more-common types of sleep loss on mood disturbance are not yet known. Therefore, the aim of this study was to examine mood disturbance in healthy adults over a week with nightly time in bed controlled at 5, 6, 7, 8 or 9 h. Participants (*n* = 115) spent nine nights in the laboratory and were given either 5, 6, 7, 8 or 9 h in bed over seven consecutive nights. Mood was assessed daily using the Profile of Mood States (POMS-2). Mixed-linear effects models examined the effect of time in bed on total mood disturbance and subscales of anger-hostility, confusion-bewilderment, depression-dejection, fatigue-inertia, tension-anxiety, vigour-activity and friendliness. There was no effect of time in bed on total mood disturbance (*F*(4, 110.42) = 1.31, *p* = 0.271) or any of the subscales except fatigue-inertia. Fatigue-inertia was higher in the 5 h compared with the 9 h time in bed condition (*p* = 0.012, *d* = 0.75). Consecutive nights of moderate sleep loss (i.e., 5–7 h) does not affect mood but does increase fatigue in healthy males.

## 1. Introduction

Mood plays a fundamental role in psychological well-being and psychopathology [1]. Patterns of mood variability are criteria for several psychiatric diagnoses (e.g., generalised anxiety, depression) [2] and can be used to predict the decline or improvement of clinical and non-clinical psychopathological symptoms [3]. The inability to regulate mood can lead to numerous problems such as reduced psychological functioning, increased depression and anxiety, and poor life satisfaction [1,4].

One factor that contributes to mood instability is sleep loss. Sleep loss has been associated with mood disturbances in both clinical [5,6] and non-clinical populations [7]. Approximately 33–45% of Australian adults report insufficient sleep duration, poor sleep quality and subsequent daytime functional impairment [8]. Additionally, between 8.3–20.5% of Australian adults experience non-clinical mood disturbances such as high psychological distress [9]. Evidently, understanding the extent to which sleep may contribute to mood variability is an important consideration.

In healthy adults, total sleep deprivation (i.e., prolonged wakefulness up to 56 h) increases symptoms of anxiety, depression, paranoia, interpersonal mistrust and hostility [10]. Similarly, sleep restriction (i.e., 4–5 h per night) lowers positive affect (e.g., vigour) and heightens negative affect (e.g., fatigue, confusion, tension, total mood disturbance) [11]. An issue arises, however, when attempting to generalise these findings to the wider population. Sleep deprivation methodologies apply stress to the human body to highlight vulnerabilities in underlying mechanisms, yet many healthy adults are not commonly exposed to sleep deprivation. Sleep restriction is a more realistic model, but only 4–5 h in bed is severe and may not reflect the low end of the general population’s sleep duration range. For this reason, moderate sleep loss (i.e., 5–7 h in bed) might be a more naturalistic approach [8].

Australian adults report an average sleep duration of 7 h per night, with 12% obtaining less than 5.5 h per night [8]. The aim of the present study was to examine the impact of different durations of time in bed (i.e., 5, 6, 7, 8 or 9 h) on mood in healthy adults over seven consecutive nights. The durations of time in bed were chosen to reflect the range habitually obtained by the adult Australian population [8]. Since the present study was conducted in a controlled environment, the impact of consecutive days in the laboratory on mood was also examined [12].

## 2. Results

### 2.1. Effect of Time in Bed on Mood

There was no main effect of time in bed on total mood disturbance, *F*(4, 110.07) = 1.43, *p* = 0.228. There was a main effect of time in bed on fatigue-inertia, *F*(4, 110.08) = 3.55, *p* = 0.009. Fatigue-inertia was higher in the 5 h condition (mean = 4.28, SD = 9.68) compared with the 9 h condition (mean = −1.58, SD = 6.47), *p* = 0.004, *d* = 0.75, large.

There were no main effects of time in bed on anger-hostility, *F*(4, 110.14) = 0.53, *p* = 0.716, confusion-bewilderment, *F*(4, 110.07) = 1.87, *p* = 0.121, depression-dejection, *F*(4, 110.10) = 1.93, *p* = 0.110, tension-anxiety, *F*(4, 110.04) = 0.86, *p* = 0.490, vigour-activity, *F*(4, 110.10) = 0.17, *p* = 0.956, or friendliness, *F*(4, 110.08) = 1.06, *p* = 0.381.

### 2.2. Effect of Study Day on Mood

**Figure 1:**
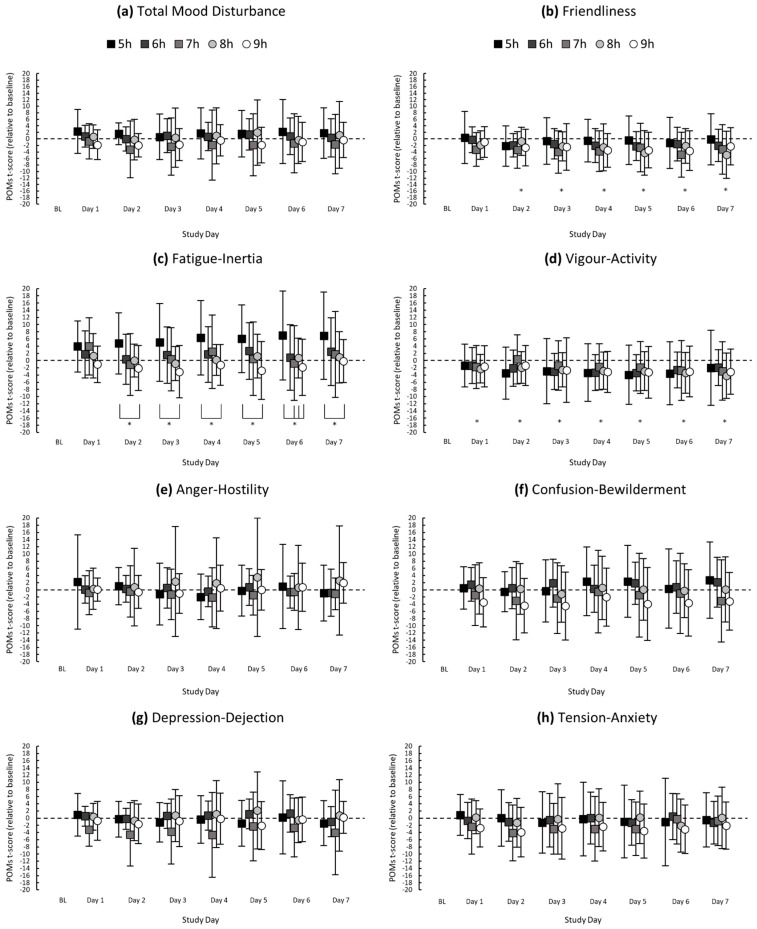
Mean POMs t-scores (relative to baseline) of (**a**) Total Mood Disturbance, (**b**) Friendliness, (**c**) Fatigue-Inertia, (**d**) Vigour-Activity, (**e**) Anger-Hostility, (**f**) Confusion-Bewilderment, (**g**) Depression-Dejection and (**h**) Tension-Anxiety for each study day. Asterisks (*) indicate a significant difference between study day and baseline; asterisks with brackets indicate a significant difference between sleep groups on the respective study day. Error bars represent standard deviations from the means.

There was no main effect of study day on total mood disturbance, *F*(7, 766.17) = 0.92, *p* = 0.492. There was a main effect of study day on fatigue-inertia, *F*(7, 766.18) = 3.27, *p* = 0.002; fatigue-inertia was higher on Day 7 compared with baseline (*p* = 0.011, *d* = 0.35, small) (see Figure 1 for all descriptive statistics).

There was a main effect of study day on vigour-activity, *F*(7, 766.18) = 8.95, *p* ≤ 0.001, and friendliness, *F*(7, 766.18) = 7.20, *p* ≤ 0.001. Compared with baseline, vigour-activity was lower on Day 1 (*p* = 0.023, *d* = 0.33; small), and vigour-activity and friendliness were lower on Day 2 (*p* = 0.018, *d* = 0.42; *p* ≤ 0.001, *d* = 0.61), Day 3 (*p* ≤ 0.001, *d* = 0.52; *p* ≤ 0.001, *d* = 0.52), Day 4 (*p* ≤ 0.001, *d* = 0.70; *p* ≤ 0.001, *d* = 0.58), Day 5 (*p* ≤ 0.001, *d* = 0.68; *p* ≤ 0.001, *d* = 0.58), Day 6 (*p* ≤ 0.001, *d* = 0.64; *p* ≤ 0.001, *d* = 0.61) and Day 7 (*p* ≤ 0.001, *d* = 0.56; *p* ≤ 0.001, *d* = 0.52), which were moderate to large differences.

There were no main effects of study day on anger-hostility, *F*(7, 766.26) = 0.33, *p* = 0.940, confusion-bewilderment, *F*(7, 766.16) = 1.42, *p* = 0.193, depression-dejection, *F*(7, 766.22) = 1.15, *p* = 0.330 or tension-anxiety, *F*(7, 766.15) = 1.96, *p* = 0.058.

### 2.3. Interactions between Time in Bed × Study Day on Mood

There was no interaction between time in bed and study day for total mood disturbance, *F*(28, 766.15) = 0.69, *p* = 0.883. There was an interaction between time in bed and study day for fatigue-inertia, *F*(28, 766.16) = 1.64, *p* = 0.020. Fatigue-inertia was higher in the 5 h condition compared with the 9 h condition on Day 2 (*p* = 0.025, *d* = 0.93), Day 3 (*p* = 0.004, *d* = 0.90), Day 4 (*p* = 0.009, *d* = 0.93), Day 5 (*p* = 0.001, *d* = 1.02), Day 6 (*p* = 0.001, *d* = 0.87) and Day 7 (*p* = 0.019, *d* = 0.76). On Day 6, fatigue-inertia was higher in the 5 h condition compared with the 7 h (*p* = 0.010, *d* = 0.68) and 8-h (*p* = 0.046, *d* = 0.69) conditions, which were all large differences.

There were no interactions between time in bed and study day for anger-hostility, *F*(28, 766.23) = 0.85, *p* = 0.695, confusion-bewilderment, *F*(28, 766.14) = 0.93, *p* = 0.572, depression-dejection, *F*(28, 766.19) = 1.11, *p* = 0.315, tension-anxiety, *F*(28, 766.13) = 0.81, *p* = 0.747, vigour-activity, *F*(28, 766.16) = 0.67, *p* = 0.907 or friendliness, *F*(28, 766.16) = 1.41, *p* = 0.077.

## 3. Discussion

The aim of the present study was to examine the effect of moderate sleep loss on subjective ratings of mood. The primary findings were that (i) fatigue was higher following consecutive nights of 5 h time in bed compared with 7–9 h time bed; (ii) most aspects of mood were not affected by the duration of time in bed; and (iii) consecutive days in the sleep laboratory reduced positive aspects of mood.

In the present study, fatigue-inertia was the only sub-scale of mood that was affected by moderate sleep loss over normal range—despite the consensus that mood is sensitive to sleep restriction or sleep deprivation [7]. Higher fatigue-inertia ratings have been reported in sleep restriction studies (i.e., 4–5 h) [10,11], and similarly, sleeping less than 5.5 h per night has been associated with increased fatigue and reduced subsequent daytime functioning in the average normal population [8]. Apart from fatigue-inertia, there was no effect of moderate sleep loss on mood. Mood is worse with both sleep restriction (i.e., 4–5 h time in bed per night) [11] and sleep deprivation (i.e., 56 h of wakefulness) [10], however these experimental manipulations are not representative of habitual sleep-wake patterns of the general population. Indeed, 80% of Australian adults report they habitually obtain between 5.5–9 h sleep per night [8]. In contrast to previous reports of impaired mood following severe sleep restriction [11] and sleep deprivation [10], moderate sleep loss over one week in the present study did not have a negative effect on participants’ mood.

Irrespective of sleep dose, seven consecutive nights in the laboratory influenced positive mood. Participants reported lower vigour-activity and friendliness during the study compared with baseline. Since there was no main effect of sleep dose on the abovementioned moods, these findings suggest the environment in the laboratory, which involves minimal exposure to natural light and reduced opportunity for exercise or socialisation, may impact mood during a multi-day sleep study. Indeed, healthy adults reported reduced happiness and increased depression following nine consecutive nights of nine hours’ time in bed in a laboratory when compared to baseline assessments [13]. Together, it seems the laboratory setting does not cause negative affect per se but may contribute to a decrease in overall positive mood.

There are advantages and disadvantages to studying mood in a laboratory setting. While keeping participants in a controlled environment isolates the effects of time in bed on mood, it also shelters participants from stressful daily events. Outside of a controlled laboratory environment, poor sleep quality has been associated with reduced positive affect and increased negative affect when responding to daily adverse events [14,15,16]. An absence of such an association in the present study implies that multiple nights of time in bed ranging from five to nine hours may not create enough vulnerability to cause an effect on mood. Given that sleep restriction in controlled environments leads to worsened mood [7,10,11] there may be a threshold of sleep loss (e.g., <5 h) below which mood may be compromised in a controlled environment without exposure to stressful events.

It is also important to note the present study had a homogenous sample of healthy young adult males. The findings may not be generalisable to females, adolescents, or older adults. Females are often excluded from studies examining the impact of sleep loss on mood to reduce confounding factors attributable to the menstrual cycle [17]. In adolescents, shorter sleep duration negatively impacts several mood states whereby this association is stronger for positive affect. Although it is estimated that nine hours per night is required for optimal mood in adolescents, the degree of sleep loss at which mood deficits occur remains unclear [18]. Less is known about the impact of sleep loss on mood in older adults. Future research should replicate the present study with females, adolescents, and older adults to determine whether mood remains unaffected by moderate sleep loss regardless of sex or age.

In conclusion, one week of moderate sleep loss (i.e., 5–7 h per night) in an environment relatively free of normal stressors does not affect mood in healthy young males but does increase feelings of fatigue. In future, it may be useful to incorporate adverse events in combination with sleep loss in a controlled laboratory environment to determine whether responses to stressful events (e.g., running late for a meeting or having a disagreement with a friend) may mediate the relationship between moderate sleep loss and mood.

## 4. Materials and Methods

### 4.1. Participants

A total of 115 healthy males aged 18–30 years (mean = 23.0, SD = 3.7) gave written informed consent to participate in the study. Exclusion criteria included smoking, excessive caffeine/alcohol use, presence or history of any psychological disorders, presence of medical or physical disorders, regular medication use, irregular sleep patterns and transmeridian travel or shift work within the last two-months. The study was approved by Central Queensland University’s Human Research Ethics Committee (H14/11-249).

### 4.2. Measures

Mood was assessed using the Profile of Mood States 2nd Edition (POMS-2) [19]. POMS-2 is a 65-item self-report scale designed to assess feelings, moods and emotions. Mood intensity is rated on a 5-point Likert scale (ranging from 0 ‘not at all’ to 4 ‘extremely’). POMS-2 includes seven subscales (Anger-Hostility, Confusion-Bewilderment, Depression-Dejection, Fatigue-Inertia, Tension-Anxiety, Vigour-Activity, Friendliness) and one summary scale (Total Mood Disturbance (sum of negative subscales minus vigour-activity)). Scores are converted to T-scores with a mean equal to 50 and a standard deviation equal to 10. The measure is deemed to have good psychometric properties (α range = 0.78–0.95) [20] and has been widely used in sleep research with healthy adults, e.g., [10,11,20,21].

### 4.3. Procedure

Participants maintained a consistent sleep/wake pattern in the week prior to the study (7–9 h of sleep per night/bedtime between 22:00 and 00:00), which was verified using self-report sleep diaries.

Participants spent nine consecutive days in the laboratory. The sleep opportunities on the first two nights (23:00–08:00) were used to familiarize participants with the sleeping environment. Participants were then randomly assigned to one of five time in bed conditions (i.e., 5, 6, 7, 8 or 9 h) for the next seven nights. In the 5, 6, 7, 8 and 9 h time in bed conditions, participants went to bed at 03:00, 02:00, 01:00, midnight and 23:00, respectively. Participants were woken at 08:00 each morning in all conditions.

Mood was assessed each day at 17:20 as per the POMS-2 manual, which defines emotional stability as no significant changes in mood over a one-week period [19]. Participants consumed standard meals for breakfast, lunch and dinner at the same time each day, and were provided with a snack after lunch and after dinner. Participants completed 3 × 10-min bouts of walking on a motorised treadmill each day at 4 km/h. In their free time, participants could read, watch movies or draw in their living rooms. Additional exercise was not permitted and interactions between participants occurred only during meals, which were served in a common dining area. Participants did not have access to their mobile phones and e-devices between 22:00 and 08:00. Internet and online phone activity (i.e., sending text messages or making phone calls) was only permitted for 20 min per day.

Participants were allocated a private bedroom, lounge room (equipped with a TV and selection of movies/television series) and bathroom. The laboratory was windowless and temperature controlled (target temperature: 21–23 °C). Light levels were set at 350 lux during wake periods, and lights were extinguished during sleep periods. In all conditions, lights were dimmed to ~10 lux from 22:00 until bedtime.

### 4.4. Statistical Analyses

Participants’ scores were expressed relative to their baseline POMs scores (i.e., POMS t-score at baseline subtracted from each score). Separate mixed-linear effects models were conducted to examine the effect of time in bed (5, 6, 7, 8 and 9 h) and study day (baseline, days 1–7) on total mood disturbance and mood subscales (anger-hostility, confusion-bewilderment, depression-dejection, fatigue-inertia, tension-anxiety, vigour-activity, friendliness). All models used Restricted Maximum Likelihood, accounted for both within and between-subject variance and specified a random effect of participant ID. Where a significant main effect was observed, pairwise comparisons were performed using a Bonferroni adjustment. Cohen’s d was calculated to determine effect size, with cut-off values of 0.2, 0.5 and 0.8 indicating small, moderate and large effect sizes, respectively [22]. Statistical analyses were completed using SPSS [23] and statistical significance was deemed as *p* ≤ 0.05. Inferential statistics are reported as F value, the degrees of freedom (in brackets) and the *p* value. Data are presented as mean ± SD.

## Data Availability

The data presented in this study are available on request from the corresponding author. The data are not currently publicly available as they are part of a larger dataset that will be used for another purpose.
